# To Take the Stairs or Not to Take the Stairs? Employing the Reflective–Impulsive Model to Predict Spontaneous Physical Activity

**DOI:** 10.3390/sports5040075

**Published:** 2017-09-29

**Authors:** Marcos Daou, Keith R. Lohse, Matthew W. Miller

**Affiliations:** 1 School of Kinesiology, Auburn University, Auburn, AL 36849, USA; mzd0046@auburn.edu; 2 CAPES Foundation, Ministry of Education of Brazil, Brasilia, DF 70047-900, Brazil; 3 Department of Health, Kinesiology, and Recreation, University of Utah, Salt Lake City, UT 84112, USA; keith.lohse@health.utah.edu

**Keywords:** reflective–impulsive model, spontaneous physical activity, self-control

## Abstract

The reflective–impulsive model (RIM) has been employed to explain various health behaviors. The present study used RIM to predict a spontaneous physical activity behavior. Specifically, 107 participants (75 females; M_age_ = 20.6 years, SD = 1.92 years) completed measures of (1) reflections about spontaneous physical activity, as indexed by self-report questionnaire; (2) impulse toward physical activity, as indexed by the manikin task; and (3) (state) self-control, as indexed by the Stroop task. The dependent variable was whether participants took the stairs or the elevator to the study laboratory. Results revealed reflections toward spontaneous physical activity positively predicted stair-taking. Further, a significant impulse toward physical activity × self-control interaction was observed. This interaction revealed that participants with high self-control who had a high impulse toward PA were more likely to take the stairs than their counterparts with a low impulse toward PA, whereas the opposite was the case for participants with low self-control. However, the impulse × self-control interaction was not significant when employing a self-report measure of trait self-control. Thus, RIM may be a good framework with which to consider spontaneous physical activity, but careful consideration must be given when examining variables within RIM (e.g., the boundary condition of self-control).

## 1. Introduction

Physical inactivity is a worldwide phenomenon, with about one-third of adults throughout the world failing to meet minimum physical activity recommendations [1]. This lack of physical activity causes an enormous burden to individuals’ health, as physical inactivity is implicated in 6–10% of all deaths from non-communicable diseases [2]. Failing to be physically active also causes tremendous economic burdens to societies, with an approximate financial cost of over $90 billion in the United States due to medical care, workers’ compensation, and productivity loss [3]. Thus, understanding the psychological determinants of physical activity is crucial.

One mode of physical activity is sports-like exercise (purposeful physical training), and another mode of physical activity is spontaneous physical activity, such as taking the stairs instead of the elevator [4]. Notably, spontaneous physical activity is a contributing component to non-exercise activity thermogenesis (NEAT), which is an important contributor to health, given its contribution to body composition [4,5]. As NEAT was not identified as a major contributor to health until the turn of the last century [5], the psychological determinants of spontaneous physical activity have received little attention. The purpose of the present study was to address this shortcoming.

A recent study investigating the psychological determinants of spontaneous physical activity is described by Cheval, Sarrazin, and Pelletier (2014) [6]. The authors examined spontaneous physical activity within the framework of the reflective–impulsive model (RIM) of behavior [7]. The RIM postulates that behavior is driven by two systems: the reflective system and the impulsive system. The reflective system guides behavior by generating plans about whether to engage in a behavior, whereas the impulsive system influences behavior by activating automatic tendencies to approach or avoid a behavior. In a clever paradigm, Cheval et al. tested whether reflections and/or impulses predicted spontaneous muscle contractions during the rest intervals between maximal voluntary contractions on a handgrip dynamometer [6]. The authors observed impulse toward physical activity positively predicted spontaneous contractions, and impulse toward sedentary behavior negatively predicted contractions, but reflections about physical activity did not predict contractions. Thus, the authors concluded that impulses, but not reflections, predicted spontaneous physical activity. 

The present study sought to build upon Cheval et al. (2014)’s compelling work in a few ways [6]. First, we sought to measure reflections about spontaneous physical activity, in contrast to Cheval et al. who assessed reflections about exercise, even though their behavior of interest was spontaneous physical activity [6]. We reasoned that by measuring reflections more closely associated with the behavior of interest (spontaneous physical activity), we would possibly be able to reveal a relationship between reflections and spontaneous physical activity. Second, we investigated whether self-control moderated the influence of reflections and impulses. We examined these interactions because it has been demonstrated that boundary conditions, such as self-control, moderate the influence of reflections and impulses on behavior [8]. For example, Xu, Li, Ding, and Lu (2014) revealed that nuclear power plant workers with high self-control had their safety behaviors driven by reflections, presumably because they were able to inhibit their impulse in order to follow through on their reflections about safety [9]. Conversely, workers with low self-control had their safety behaviors guided by impulses, presumably because they were unable to inhibit their impulse about safety. Finally, we sought to extend Cheval et al.’s work by showing that the RIM is effective for predicting another spontaneous physical activity behavior, specifically whether participants took the stairs or the elevator to our study laboratory [6]. Based on the extant literature [6,9], we predicted participants with high self-control would have their stair/elevator choice predicted by their reflections about spontaneous physical activity, whereas participants with low self-control would have their choice predicted by their impulse toward physical activity. Contrary to predictions, reflections toward spontaneous physical activity positively predicted stair-taking irrespective of self-control. As predicted, the relationship between impulse toward physical activity and stair-taking was moderated by self-control. This moderation revealed the unexpected finding that participants with high self-control who had a high impulse toward physical activity were more likely to take the stairs than their counterparts with a low impulse toward physical activity, whereas the opposite was the case for participants with low self-control. However, the reliability of this moderation depended on whether a behavioral measure of state self-control or a self-report measure of trait self-control was used. In the latter case, self-control did not moderate a relationship between impulse toward spontaneous physical activity and stair-taking.

## 2. Materials and Methods

All experimental design, data collection, and data analysis plans were preregistered and can be found at https://aspredicted.org/public/204787165.pdf. 

An a priori power calculation was conducted with G*Power 3.1 [10]. We assumed a small effect size (f^2^ = 0.1), α = 0.05, and β = 0.8. The power analysis was for a linear regression with the goal of detecting a significant change in the model when adding two significant interaction terms. Thus, the predicted effect size was based on a pseudo-*r*-square. We had two tested predictors: the interaction between self-control and reflections about spontaneous physical activity as well as the interaction between self-control and impulse toward physical activity. These predictors were added in the final step of a regression model, which included the following predictors (based on Cheval et al., 2014 and Cheval, Sarrazin, Isoard-Gautheur, Fadel, & Friese, 2014 [6,11]) in prior steps: age, sex, typical physical activity, reflections about spontaneous physical activity, impulse toward spontaneous physical activity, and self-control. The power calculation yielded a sample size of 100, but we decided to collect data from 120 participants to account for incomplete datasets. In the end, 127 participants, who were recruited from university classes and by word-of-mouth, signed-up for and attended the data collection. Participants were compensated with class credit and entry into a raffle for a monetary reward. We excluded data from 20 participants for the following reasons: failure to report typical physical activity (*n* = 9); reporting over 4 h/day of moderate–vigorous physical activity (*n* = 1); failure to complete test of impulse toward physical activity (*n* = 2); failure to complete test of self-control (*n* = 5); failure to complete questionnaire about reflections on spontaneous physical activity (*n* = 1); and thinking the stairs/elevator choice was part of the study (*n* = 2). Thus, the final sample included 107 participants (75 females; M_age_ = 20.6 years, SD = 1.92 years).

To measure reflections toward spontaneous physical activity, we asked participants to complete visual analog scales. Specifically, we asked participants to draw a vertical line across a horizontal line, anchored by completely disagree and completely agree, to indicate how much they concurred with statements about their recent and present intentions for engaging in 24 different behaviors, ranging from attending a party to traveling. Among these behaviors, we included the following spontaneous physical activities:

- walk/bike to destinations, when they are accessible;
- when given the choice, stand instead of sit;
- always take the parking spot closest to the destination (reverse scored).

Statements related to the other 21 behaviors were included in attempt to mask the purpose of the study.

To examine the internal consistency of responses about the behaviors, we conducted a Chronbach’s alpha test, which yielded a value of 0.449 (correlations among the responses are shown in Table 1). Despite the low Chronbach’s alpha, we averaged across responses about the behaviors to create a single measure of reflections toward spontaneous physical activity for our primary analysis. This decision was based on our a priori plan of analysis as well as our desire to avoid conducting separate regressions wherein each regression included reflections about a specific spontaneous physical activity behavior. Additionally, despite the low correlation, positive scores on each measure indicate greater intentions toward a spontaneous physical activity. 

To measure impulse toward physical activity, we employed the manikin task described by Cheval, Sarrazin, Isoard-Gautheur et al. (2014) [11]. In this task, a stick-figure human (manikin) appears at either the top or bottom of a computer monitor screen, followed by an image of stick-figure human engaged in physical activities (e.g., cycling) or sedentary activities (reclining in a chair). Participants are asked to use a computer keyboard to move the manikin toward or away from the image, depending on the task condition (physical activity/sedentary) currently being performed. In the physical activity condition, participants are instructed to move toward physical activity images and away from sedentary images, whereas they are instructed to do the opposite in the sedentary condition. Participants’ response time to move the manikin is recorded. Participants complete 12 practice trials and 64 test trials in each condition. For further task details, please see Cheval, Sarrazin, Isoard-Gautheur et al. [11]. 

Trials on which participants responded incorrectly, below 150 ms, or above 1500 ms were excluded from analysis [6,11]. Next, each participant’s impulse toward physical activity was determined by subtracting their median response time to (correctly) move the manikin toward physical activity images from their median response time to (correctly) move the manikin away from physical activity images. Median, as opposed to mean, response time was employed based on Cheval, Sarrazin, and Isoard-Gautheur et al. [11]. Thus, positive scores indicate an impulse toward physical activity, and negative scores indicate an impulse to avoid physical activity. Additionally, each participant’s impulse toward sedentary behavior was calculated by subtracting median response time to move the manikin toward sedentary images from their median response time to move the manikin away from sedentary images, and this impulse toward sedentary behavior score was used for secondary analyses.

To measure self-control, we employed a Stroop task, which participants performed on a computer [12]. Specifically, participants were instructed to respond to the color with which text was presented on a computer screen as quickly as possible by pressing color-labeled keys on a computer keyboard (red on V, blue on B, green on N, and yellow on M keys). On each trial, the text remained on the screen until participants responded, and then a fixation cross appeared on the screen for 1000 ms, after which the next trial began.

To facilitate stimulus–response compatibility, participants first completed a practice block consisting of 100 neutral trials. Each neutral trial contained a series of Xs (e.g., XXXX) in one of the four colors. Next, participants completed a practice block consisting of 30 trials, 15 of which were congruent and 15 of which were incongruent (congruent and incongruent trials were interspersed randomly). Each congruent trial consisted of text spelling out the color of the text (e.g., BLUE in blue color), whereas each incongruent trial consisted of text spelling out a different color from that of the text (e.g., BLUE in red color). Incongruent trials required participants to inhibit their tendency to respond based on the color written out in the text. After the 30-trial practice block, participants completed a test block of 144 trials, which included randomly ordered neutral, congruent, and incongruent trials. After removing trials with responses below 150 ms or above 1500 ms, median response times for congruent and incongruent trials were determined. Then, the Stroop interference score was calculated by subtracting median incongruent response time from median congruent response time. Thus, higher Stroop interference scores indicated higher self-control [9]. In addition to the Stroop interference score, which is a behavioral measure of state self-control, we also assessed self-reported trait self-control with the Tangney Self-Control Scale (SCS; [13]). The SCS includes items such as “I have a hard time breaking bad habits” and had good reliability (Chronbach’s α = 0.785). It has been suggested that behavioral measures of state self-control and self-report measures of trait self-control reflect different elements of self-control, which is evidenced by the failure of these measures to reliably correlate [14]. As a secondary analysis, we decided to substitute the SCS for the Stroop interference score in whichever model predicted stair-taking better: the model including either impulse toward physical activity and sedentary behavior, or the model including just impulse toward physical activity.

Based on Cheval, Sarrazin, and Pelletier (2014) [11], we employed the International Physical Activity Questionnaire (IPAQ; [15]) to index participants’ typical physical activity in order to control for this variable. Specifically, participants reported the number of days during the past week in which they engaged in moderate or vigorous physical activity, and the amount of time they usually spent engaged in the activity on a day. The products of the number of days per week and the amount of time usually spent engaged in the activity were calculated for moderate and vigorous physical activity, and then the products were summed to create the typical physical activity variable. 

An experimenter met each participant at the entrance to the bottom-floor of a three-floor building between 12 and 5 p.m. Only participants who were unfamiliar with the building were recruited so as to avoid having participants take the stairs/elevator out of a building-specific habit. Next, the experimenter walked the participant through a hallway to the hallway’s end, where an elevator and stairwell were located directly across from one another. Then, the experimenter touched their front pants pocket and told the participant, “I’m sorry, I can’t find my card. I think I forgot my ID card to open the lab’s door in my car… I’ll grab the card and meet you at the third floor in a minute… You can go ahead and take the stairs or the elevator to the top floor; they are equally fast. See you in a minute.” The researcher then turned back and started running toward building’s exit to the parking lot. The researcher turned their head back and noted whether the participant decided to take the stairs or elevator. A few minutes later, the experimenter met the participant in front of the laboratory with their ID card in hand, then swiped their ID card to open the laboratory door. In the lab, participants completed informed written consent (Auburn University Institutional Review Board Protocol 15-306 EP 1507). Next, participants completed the manikin task and the Stroop task, the order of which was counterbalanced across participants. Then, participants completed the visual analog scales measuring reflections toward spontaneous physical activity, IPAQ, and SCS. Next, participants responded to questions about whether they had access to a bicycle and/or car, as well as reported their height and weight for body mass index (BMI) calculation. As an exploratory analysis, we were interested in whether BMI would predict stair-taking; if it did, then we planned to control for it. Additionally, we planned to statistically control for access to a bicycle or car, if such access significantly affected how participants responded to our reflections toward a spontaneous physical activity questionnaire. Before participants left the lab, they were debriefed and asked if they suspected the stairs/elevator choice was part of the study.

The dependent variable of whether participants took the stairs or elevator to the laboratory was predicted with a multi-step binary logistic regression. The first step of the regression included sex, age (mean centered), typical physical activity (mean centered), and bicycle access (which significantly affected participants’ reflections toward spontaneous physical activity, *p* = 0.021). Access to a car did not significantly affect participants’ reflections toward spontaneous physical activity (*p* = 0.742), and BMI did not predict stair-taking (*p* = 0.404). The second step of the regression added reflections toward spontaneous physical activity (z-score standardized), impulse toward physical activity (z-score standardized), and self-control (z-score standardized). The final step of the regression added the reflections toward spontaneous physical activity × self-control interaction and the impulse toward physical activity × self-control interaction. Alpha levels were set to 0.05. 

## 3. Results

**Figure 1:**
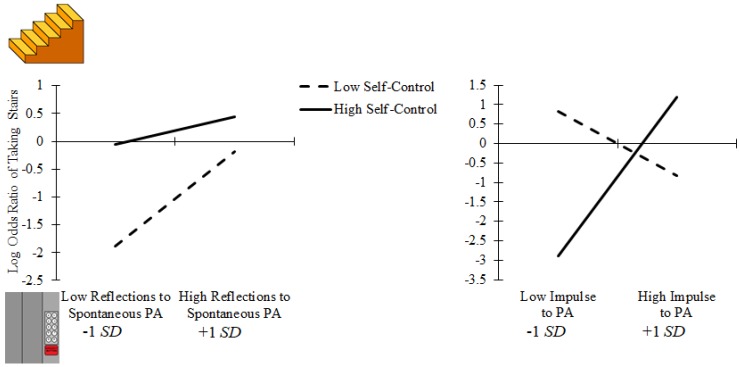
(**Left Panel**) Log odds ratio of taking the stairs as a function of reflections toward spontaneous physical activity and self-control. Greater reflections toward spontaneous physical activity increased the odds of taking the stairs. (**Right Panel**) Log odds ratio of taking the stairs as a function of impulse toward physical activity and self-control. A significant interaction between impulse toward physical activity and self-control revealed participants with high self-control who had a high impulse toward physical activity had greater odds of taking the stairs than their counterparts with a low impulse toward physical activity, whereas the opposite was the case for participants with low self-control.

The regression revealed three participants whose data excessively influenced the regression model (Cook’s distances ≥ 1.09; [16]), so these participants’ data were removed. If these participants remain in the analysis, the only primary result that changes in terms of statistical significance is that reflections toward spontaneous physical activity becomes marginally nonsignificant (*p* = 0.079) in the third step of the regression model. Regression results are shown in Table 2. The first step of the regression revealed neither sex, age, bike access, nor typical physical activity significantly affected the odds ratio of taking the stairs (*ps* ≥ 0.179). The second step of the regression showed that neither sex, age, bike access, typical physical activity, impulse toward physical activity, nor self-control significantly affected the odds ratio of taking the stairs (*ps* ≥ 0.327). However, greater reflections toward spontaneous physical activity did significantly increase the odds ratio (1.74) of taking the stairs (*p* = 0.016). The third step of the regression showed that neither sex, age, bike access, typical physical activity, impulse toward physical activity, self-control, nor the reflections toward spontaneous physical activity × self-control interaction significantly affected the odds ratio of taking the stairs (*ps* ≥ 0.102). However, greater reflections toward spontaneous physical activity did significantly increase the odds ratio (1.73) of taking the stairs (*p* = 0.027). Additionally, the impulse toward physical activity × self-control interaction significantly influenced the odds ratio (4.20) of taking the stairs (*p* = 0.004). This interaction revealed that impulse predicted stair-taking for those participants with low self-control and those participants with high self-control (see Figure 1). Low and high self-control are relative to the sample. In the figure, this is represented as −1 SD and +1 SD relative to M. Specifically, participants with high self-control who had a high impulse toward physical activity were more likely to take the stairs than their counterparts with a low impulse toward physical activity, whereas the opposite was the case for participants with low self-control. Notably, the results did not change in terms of statistical significance when adding impulse toward sedentary behavior or the impulse toward sedentary behavior × self-control interaction to the model (reflections toward spontaneous physical activity: odds ratio = 1.68, *p* = 0.038; impulse toward physical activity × self-control: odds ratio = 4.26, *p* = 0.013). Further, this model less accurately predicted stair-taking in comparison to the model without impulse toward sedentary behavior and impulse toward sedentary behavior × self-control (70.2% vs. 73.1%).

Finally, we substituted our measure of self-reported state self-control (SCS) for the Stroop interference score (these two measures were not significantly correlated (*p* = 0.576), in accord with Allom et al. (2016) [14]). Results from the models with SCS instead of Stroop interference score differed in terms of statistical significance. Specifically, impulse toward physical activity × self-control was not significant (*p* = 0.594). Reflections toward spontaneous physical activity remained significant (odds ratio = 1.59, *p* = 0.039).

## 4. Discussion

The present study sought to expand upon a recent effort to use RIM to predict spontaneous physical activity [6], specifically stair-taking. In particular, the present study employed a reflective predictor closely associated with stair-taking by measuring reflections toward spontaneous physical activity. Additionally, the present study examined whether the boundary condition of self-control moderated (1) the relationship between reflections toward spontaneous physical activity and stair-taking and (2) impulse toward physical activity and stair-taking. In accord with RIM, we predicted both moderations would be significant, such that reflections toward spontaneous physical activity would reliability predict stair-taking exclusively for participants high in self-control, and impulse toward physical activity would reliability predict stair-taking exclusively for participants low in self-control. Results were largely in contrast with predictions. Specifically, results suggest reflections toward spontaneous physical activity may predict stair-taking, and the relationship between impulse toward physical activity and stair-taking may be moderated by self-control. However, this moderation revealed the unexpected finding that participants with high self-control who had a high impulse toward physical activity were more likely to take the stairs than their counterparts with a low impulse toward physical activity, whereas the opposite was the case for participants with low self-control. Further, the reliability of this moderation may depend on how self-control is measured. In particular, a behavioral measure of state self-control revealed it a reliable moderator, whereas a self-report measure of trait self-control did not. 

The result that reflections toward spontaneous physical activity predicted stair-taking, irrespective of state or trait self-control, is contrary to predictions but not completely unexpected. Indeed, we speculated that one of the reasons Cheval et al. (2014) did not observe reflections toward physical activity predicted spontaneous physical activity was that the authors measured reflections toward exercise [6]. Thus, we may have detected a relationship between reflections and stair-taking because we measured reflections toward spontaneous physical activities potentially associated with stair-taking. However, an important caveat to this result is that our measures of reflections toward spontaneous physical activities exhibited poor internal reliability (consistency) with a Chronbach’s alpha of 0.449. This poor consistency brings into question the reliability of the relationship between spontaneous physical activity and stair-taking, but the poor consistency is also interesting in its own right. Specifically, the poor consistency suggests particular spontaneous physical activities may not be highly correlated. If this is the case, then the relationships between reflections and impulses toward spontaneous physical activity, as well as the moderating boundary conditions, may be dependent on the particular activities about which reflections are made and the particular activity serving as the outcome variable. From a theoretical perspective, this dependency on the particular spontaneous physical activities may make sense. Specifically, although genetic factors may broadly predict various types of spontaneous physical activities, some activities are likely unique to environmental factors (e.g., whether there are bike lanes/sidewalks near one’s residence) [4]. Of course, the consistency with which individuals engage in a particular spontaneous physical activity (e.g., taking the stairs) may also vary on a situation-by-situation basis. For example, a person’s legs may be fatigued from having recently lifted weights with their legs, thus causing them to take the elevator instead of taking the stairs, as they typically would. Thus, our spontaneous physical activity variable may be somewhat unstable. 

The unexpected result that participants with high self-control who had a high impulse toward physical activity were more likely to take the stairs than their counterparts with a low impulse toward physical activity, whereas the opposite was the case for participants with low self-control is inexplicable to us. However, an important limitation of our measure of impulse toward physical activity is worth noting. Specifically, we measured impulse toward physical activity, not spontaneous physical activity. It is possible measuring impulse toward spontaneous physical activity could have revealed a more sensible relationship between this variable and spontaneous physical activity. Importantly, it is worth noting that the interaction between impulse toward spontaneous physical activity and self-control was only significant when a behavioral measure of state self-control was employed, not when a self-reported measure of trait self-control was used. These discrepant findings are not surprising when considering behavioral measures of state self-control may be uncorrelated with self-report measures of trait self-control. For example, Allom, Panetta, Mullan, and Haggar (2016) observed a nonsignificant relationship (r = −0.033, *p* > 0.05) between the very same measures of state and trait self-control employed in the present study, wherein the measures were also not significantly correlated (r = 0.048, *p* > 0.05) [14]. Notably, RIM proposes both state and trait factors can serve as boundary conditions, and the present experiment suggests both types of factors should be considered as they may uniquely interact with reflections and impulses.

Spontaneous physical activity is an important contributor to health, so understanding its psychological determinants is crucial [4,5]. The present study is one of a couple of recent efforts to attempt to use RIM to reveal the determinants of spontaneous physical activity [6]. A unique contribution of the present study was the consideration of a boundary condition (self-control) in predicting spontaneous physical activity from RIM. The present study observed unexpected (and perhaps inexplicable) effects of the boundary condition, but boundary conditions are a critical part of RIM. Since RIM has been employed to explain numerous health behaviors and boundary conditions are an important component of RIM, we encourage future research efforts employing RIM and various boundary conditions to predict spontaneous physical activity. As researchers use RIM to investigate spontaneous physical activity, we suggest careful consideration of the way in which reflections, impulses, and boundary conditions are indexed, as well as the measurement of different spontaneous physical activities, which may not be highly correlated within individuals.

## Figures and Tables

**Table 1:** Relationships among reflections toward spontaneous physical activities.

Correlation Coefficients Among Reflections Toward Spontaneous Physical Activities			
1. Walk/Bike vs. Drive		2. Stand vs. Sit	3. Don’t Park as Close as Possible to Destination
1	-	0.192 ^*^	0.054
2		-	0.372 ^**^

^*^ *p* ≤ 0.05, ^**^ *p* ≤ 0.01.

**Table 2:** Results from primary regression models predicting stair-taking. Statistically significant predictors are in boldface.

**Model 1: Probability of Taking Stairs ~ Sex + Age + Bike Access + Typical PA**				
	χ^2^	df	Percent Correctly Classified	
Regression	2.38	4	57.7	
Coefficients	β	*SE*	*OR*	*p*-value
Constant	−0.377	0.280	0.686	0.179
Sex	−0.048	0.224	0.953	0.829
Age	0.067	0.105	1.07	0.522
Bike Access	0.472	0.405	1.60	0.244
Typical PA	0.000	0.001	1.00	0.661
**Model 2: Probability of Taking Stairs ~ Sex + Age + Bike Access + Typical PA +** **Impulse to PA + Reflections to PA + Self-Control**				
	χ^2^	df	Percent Correctly Classified	
Regression	9.24	7	66.3	
Coefficients	β	*SE*	*OR*	*p*-value
Constant	−0.290	0.296	0.748	0.327
Sex	−0.140	0.241	0.870	0.562
Age	0.054	0.112	1.06	0.629
Bike Access	0.274	0.427	1.32	0.521
Typical PA	0.000	0.001	1.00	0.995
Impulse to PA	0.138	0.217	1.15	0.523
**Reflections to PA**	**0.552**	**0.230**	**1.74**	**0.016**
Self-Control	0.053	0.261	1.05	0.839
**Model 3: Probability of Taking Stairs ~ Sex + Age + Bike Access + Typical PA +** **Impulse to PA + Reflections to PA + Self-Control +** **Impulse to PA × Self-Control + Reflections to PA × Self-Control**				
	χ^2^	df	Percent Correctly Classified	
Regression	23.0	9	73.1	
Coefficients	β	*SE*	*OR*	*p*-value
Constant	−0.422	0.324	0.656	0.192
Sex	−0.169	0.256	0.845	0.510
Age	0.055	0.120	1.06	0.646
Bike Access	0.129	0.468	1.14	0.783
Typical PA	0.000	0.001	1.00	0.830
Impulse to PA	−0.425	0.347	0.654	0.221
**Reflections to PA**	**0.547**	**0.248**	**1.73**	**0.027**
Self-Control	0.615	0.376	1.85	0.102
**Impulse to PA × Self-Control**	**1.43**	**0.504**	**4.20**	**0.004**
Reflections to PA × Self-Control	−0.301	0.373	0.740	0.421

**Notes:** Stairs = 1, Elevator = −1; Male = −1, Female = 1; Age and Typical PA (Physical Activity) are median-centered; Impulse to PA, Reflections to PA, and Self-Control are standardized (z-score).

## Supplementary Materials

The spreadsheet used for statistical analysis is available online at http://www.mdpi.com/2075-4663/5/4/75/s1.
